# Factors associated with re-admission in the year after acute postpartum psychiatric treatment

**DOI:** 10.1007/s00737-022-01255-3

**Published:** 2022-08-25

**Authors:** Billie Lever Taylor, Angela Sweeney, Laura C. Potts, Kylee Trevillion, Louise M. Howard

**Affiliations:** 1 Section of Women’s Mental Health, Institute of Psychiatry Psychology and Neuroscience, King’s College London, London, UK; 2 Health Services and Population Research Department, Institute of Psychiatry, Psychology and Neuroscience, Kings College London, London, UK; 3 Department of Biostatistics and Health Informatics, Institute of Psychiatry, Psychology and Neuroscience, King’s College London, London, UK

**Keywords:** Perinatal mental health, Acute psychiatric care, Childhood trauma, Parent–infant bonding, Mothers

## Abstract

**Purpose:**

To examine factors associated with being re-admitted in the year after discharge from acute postpartum psychiatric treatment.

**Methods:**

Secondary data analysis of information collected from mothers who were admitted to acute psychiatric services in the year after childbirth between 2013 and 2017. We carried out univariable analyses and multivariable hierarchical logistic regression to examine risk factors for women’s re-admission to acute psychiatric care (inpatient or community crisis care) in the year following discharge.

**Results:**

Sixty-seven (24.1%) of 278 women were re-admitted in the year after discharge from acute care; the median number of days to re-admission was 86 (IQR 35–214), and women who were re-admitted accessed a median of two further acute services (IQR 1–3). In adjusted analyses, reporting a history of childhood trauma (aOR 1.02; 95% CI 1.00- 1.03, p = 0.036), a higher level of difficulties in the mother–infant bond (aOR 1.03; 95% CI 1.01–1.06, p = 0.009) and younger age (aOR 0.95; 95% CI 0.90–1.00, p = 0.066) were associated with re-admission.

**Conclusion:**

This study confirms that the role of childhood adverse experiences on mental health is relevant for outcomes in women experiencing acute postpartum psychiatric episodes. Ongoing parent–infant bonding difficulties are also independently associated with re-admission. Perinatal mental health services therefore need to offer evidence-based interventions to address histories of trauma and to support parent–infant bonding to optimise mental health in women following discharge from acute psychiatric services. However, further research is needed to explore what other factors, not measured in our study, are also influential to re-admission.

## Introduction

It is well established that women are at an increased risk of being hospitalised for a severe psychiatric diagnosis, such as puerperal psychosis, severe depression, schizophrenia or bipolar disorder, in the postpartum period (Kendell et al. 1987; Munk-Olsen et al. 2006). However, there has been very little research into what happens to women after discharge from acute services for postpartum psychiatric treatment.

In a recent study, ‘Effectiveness of Services for Mothers with Mental Illness’ (ESMI), conducted with women discharged from acute postpartum psychiatric care across England and Wales, Howard et al. (2022a) found that around a quarter of women were re-admitted in the following year. This indicates ongoing severe difficulties after an acute care admission, at a time when the developing relationship between mother and infant remains crucial for maternal and child outcomes (Stein et al. 2014).

These findings replicate the only other research to our knowledge that has examined re-admission and mother–infant outcomes: a small study of 45 women admitted to a three-bed specialist mother and baby unit (MBU) in New Zealand found that, at three months post-discharge, nearly a third of mothers (31%) were experiencing a significant deterioration, 17% had been re-admitted to inpatient care, and 83% were rated as having ongoing difficulties in parent–infant bonding (Wright et al. 2020).

These studies suggest women may have continuing unmet needs following acute postpartum admissions, but it is not yet known which factors are associated with re-admission. This is important to understand because outcomes for women and their babies are poorer when women experience ongoing mental health difficulties and have repeated admissions (Stein et al. 2014; Netsi et al. 2018). Our goal in this study, therefore, was to examine factors associated with re-admission following discharge from acute postpartum psychiatric treatment in order to increase our understanding of which women are at high risk of re-admission and to explore their needs.

## Method

### Study setting and participants

Participants in this study were from the ESMI study, a quasi-experimental study of women accessing different types of acute psychiatric services in the first year after childbirth: see Trevillion et al. (2019) for the study protocol including full details on recruitment, data collection, outcome measures, and design. Postpartum women (n = 279) who had been admitted to a psychiatric MBU, general acute ward or crisis resolution team (CRT) (or any combination of these) in the first year after childbirth (between 2013 and 2017), were recruited from 42 diverse mental healthcare provider organisations across England and Wales.

MBUs (which are also available in parts of Europe, Asia, North America, and Australia) are recommended as the ‘gold standard’ for women with acute postpartum psychiatric diagnoses, enabling women to be co-admitted to hospital with their babies and supported by a team of specialist clinicians (Royal College of Psychiatrists. 2021). In some cases, however, for example if there is no MBU nearby (as was the case in many areas of the UK at the time of this study), women are instead admitted to general psychiatric wards, usually involving separation from their baby. As an alternative to a hospital admission (or in addition), women with acute diagnoses may receive intensive home treatment from a multidisciplinary CRT.

Women were excluded if they lacked capacity to consent, were using an acute service ‘prophylactically’ (e.g. for a statutory parenting assessment), or if their baby had been permanently removed from their care prior to the admission.

Women were interviewed one month after discharge from acute care, with interpreters where needed. Data collection was via researcher-administered questionnaires, observations of parent–infant interactions, and reviews of clinical notes. With consent, at one year post-discharge from acute services, 278/279 women were successfully followed up via a telephone interview and/or review of clinical case notes. NHS ethics approval was obtained (reference: 14/LO/0765), and all participants provided written informed consent.

### Measures

#### Outcome

The outcome was re-admission to acute psychiatric care (MBU, general acute ward, or CRT) by one year after discharge from an initial acute postpartum admission (binary variable; yes/no).

#### Potential risk factors for re-admission

#### Socio-demographics

Key socio-demographic data in our analyses included maternal age (continuous variable), maternal ethnicity (categorised as: White; Black African, Caribbean or Black British; Asian or Asian British; Mixed ethnicity; or Other), education (categorised as whether or not the mother had attended ‘further education’ beyond age 16: yes/no), living alone at one month post-discharge (yes/no), and primiparity (yes/no).

#### Developmental and interpersonal abuse

Women completed the Child Trauma Questionnaire (CTQ), a validated 28-item self-report Likert scale measuring sexual, emotional, and physical abuse and neglect in childhood. CTQ subscale scores range from 5 to 25 (with recommended cut-offs for moderate/severe trauma) (Bernstein and Fink 1998). Total scores range from 25 to 125.

The Composite Abuse Scale (CAS) (Hegarty et al. 2005), a validated 30-item measure of interpersonal partner violence, was also administered. Items are rated from 0 = ’never’ to 5 = ‘daily’. Scores of 3 + indicate partner violence. This scale was administered at one month post-discharge but was modified to collect data covering: (1) the 12 months prior to admission and (2) the point of discharge to one month post-discharge.

#### Clinical factors

We examined clinical factors, including whether the mother had other psychiatric admissions in the two years preceding the index postpartum admission (yes/no); whether the mother’s first contact with mental health services was at under age 18 (yes/no); mother’s highest level of care received^1^ (MBU, acute ward, or CRT); maternal substance use^2^ (yes/no); clinical diagnosis of: schizophrenia (yes/no), bipolar disorder (yes/no), depression (yes/no), or personality disorder (yes/no) (diagnosis was based on International Classification of Diseases (ICD-10) standard diagnostic classifications as recorded in clinical case notes at the time of the index postpartum admission, grouped using the ICD-10 hierarchy; see Howard et al. (2022b) for more detail; and whether the mother was detained under the Mental Health Act (Department of Health. 2015) during her admission (yes/no).

#### Unmet needs post-discharge

Women reported their unmet health and social care needs at one month post-discharge using the researcher-administered Camberwell Assessment of Need for Mothers (Short Version) (CAN-M(S)), a 26-item validated questionnaire (Howard et al. 2008). Each item is scored on a scale from 0–2, and items are summed to generate a total number of ‘met’ and/or ‘unmet’ needs.

#### Parent–child relationship difficulties post-discharge

Mother–child interactions were assessed at one month post-discharge in a 3-min video clip during play at home. Videos were rated using the Child and Adult Relationship Experimental Index (CARE-Index (Crittenden 2003)) across four scales (maternal sensitivity, maternal unresponsiveness, infant cooperation, and infant passivity), each ranging from 0 to 14. Higher scores of sensitivity on the mother scale and cooperativeness on the infant scale indicate stronger dyadic synchrony, while higher scores for maternal unresponsiveness or infant passivity indicate weaker dyadic synchrony. While Crittenden (2003) cautions against applying cut-offs rigidly to this index, it has been suggested that, on the maternal sensitivity scale, a score of 7 + is ‘adequately’ sensitive (Crittenden 2003; Parfitt et al. 2013).

Women also completed the 25-item self-report Postpartum Bonding Questionnaire (PBQ) about their feelings and attitudes towards their child (Brockington et al. 2001). A systematic review found this was the only self-report measure of the parent–infant relationship to demonstrate sufficient evidence for structural validity, internal consistency, and reliability with high quality of evidence (Wittkowski et al. 2020). Higher scores indicate increased bonding difficulties, and a cut-off score of > 25 has also been suggested to indicate significant bonding difficulties (Brockington et al. 2006).

### Data analysis

Quantitative data were analysed using STATA version 17.

Women’s characteristics were described both overall and by re-admission status. Continuous measures were summarised using the mean and standard deviation, or median and interquartile range for variables that were skewed. Categorical measures were summarised using tallies and percentages.

Univariable analyses were undertaken to assess associations between re-admission and potential risk factors. Between-group comparisons of continuous data were made using the independent samples *t* test or nonparametric Mann–Whitney U test where data were not normally distributed. Pearson’s Chi-square (*χ*^*2*^) test of the independence of variables was used for the analysis of categorical data or Fisher’s exact test where cell sizes were small (expected frequency < 5).

Hierarchical logistic regression was used in follow-up analyses to examine which socio-demographic, clinical, or wider factors that had shown between-group differences in the univariable analyses (p < 0.1) were associated with re-admission in the year after discharge, adjusting for covariates. We first assessed each variable individually to establish the unadjusted magnitude of effect. We then built a hierarchical logistic regression model (using the ‘nestreg’ function in Stata), inputting variables in the following steps: (Step 1) background maternal factors (age, education, childhood trauma); (Step 2) clinical variables (previous admissions in past 2 years); (Step 3) continuing difficulties post-discharge (score on postpartum bonding questionnaire).

#### Missing data

We had complete data (n = 279) for all socio-demographic and clinical variables included, with the exception of whether the woman had contact with services before age 18 (9/279 responses missing). We also had complete data for unmet needs (CAN-M(S)). Re-admission data were available for 278/279 participating women. We imputed mean scores where <  = 20% of items were missing on CAS subscales, CTQ subscales, or on the PBQ.

We conducted a complete case analysis, including participants with data on all variables in the analyses. Our final hierarchical logistic regression model included 251 of 278 (90%) participating women. As a sensitivity analysis, we repeated our logistic regression model using multiple imputation with chained equations (MICE) to replace missing data on covariates and the outcome variable (only one woman had missing *outcome* data). We assumed that data were missing at random (i.e. that missing data were associated with observed data) and imputed 50 data sets, using all variables included in our logistic regression analyses as well as auxiliary variables (ethnicity and living alone status). We re-ran analyses according to Rubin’s rules (Sterne et al. 2009).

## Results

### Women’s characteristics by re-admission status

Sixty-seven of the 278 women (24.1%) were re-admitted to an acute psychiatric service (MBU, acute ward or CRT) by one year post-discharge, with 37 (55%) of these women having an inpatient admission (previous analyses of ESMI data found no evidence of a difference in re-admission rate by type of service (Howard et al. 2022a). Between them, these women accessed acute psychiatric services 150 times in the year after discharge, ranging from 1 acute service accessed (31 women) to 12 acute services accessed (1 woman), with a median of 2 acute services accessed per woman. Sixty-seven of the 150 (45%) re-admissions were inpatient admissions. The median number of days to first re-admission was 86 (interquartile range 35–214).

Table 1 shows characteristics of women by re-admission status, along with univariable analyses of associations between re-admission and potential risk factors. There was weak evidence that women who were re-admitted were younger (mean age 30 versus 32 years; t = 1.76, p = 0.080). They were also less educated (28.4% versus 16.6% with no further education; χ2 = 4.50, p = 0.034) and more likely to have had a previous admission in the past two years (26.9% compared with 14.2%; χ2 = 5.69; p = 0.017).

**Table 1:** Characteristics of women who were or were not re-admitted following discharge from acute postpartum psychiatric care

**Variable **	**N**	**Level**	**Total **	**Re-admitted **	**Not re-admitted**	**Significance test**
**Age (at time of consent)**	**278**	**Mean (SD)**	**31.5 (6.0)**	**30.4 (0.7)**	**31.9 (0.4)**	**t=1.76; p=0.080**
Ethnicity	278	White	212 (76.3)	52 (77.6)	160 (75.8)	χ^2^=4.75; p=0.335^1^
		Black African/Caribbean/Black British	20 (7.2)	5 (7.5)	15 (7.1)	
		Asian or Asian British	24 (8.6)	3 (4.5)	21 (10.0)	
		Mixed Ethnicity	11 (4.0)	2 (3.0)	9 (4.3)	
		Other Ethnicity	11 (4.0)	5 (7.5)	6 (2.8)	
**Further education**	**278**	**Yes** **No**	**224 (80.6)** **54 (19.4)**	**48 (71.6)** **19 (28.4)**	**176 (83.4)** **35 (16.6)**	**χ**^**2**^**=4.50; p=0.034**
Living alone (at one-month post-discharge)	278	Yes No	47 (16.9) 231 (83.1)	15 (22.4) 52 (77.6)	32 (15.2) 179 (84.8)	χ^2^=1.89; p=0.169
Any other children	278	Yes	125 (45.0)	35 (52.2)	90 (42.7)	χ^2^=1.89; p=0.169
		No	153 (55.0)	32 (47.8)	121 (57.4)	
Substance use	278	Yes No	30 (10.8) 248 (89.2)	8 (11.9) 59 (88.1)	22 (10.4) 189 (89.6)	χ^2^=0.12; p=0.728
Contact with services <age 18	269	Yes	53 (19.7)	16 (24.6)	37 (18.1)	χ^2^=1.31; p=0.253
		No	216 (80.3)	49 (75.4)	167 (81.9)	
**Previous admissions in last 2 years**	**278**	**Yes**	**48 (17.3)**	**18 (26.9)**	**30 (14.2)**	**χ**^**2**^**=5.69; p=0.017**
		No	230 (82.7)	49 (73.1)	181 (85.8)	
Detention under Mental Health Act for current admission	278	Yes	80 (28.8)	21(31.3)	59 (28.0)	χ^2^=0.28; p=0.594
		No	198 (71.2)	46 (68.7)	152 (72.0)	
**Personality disorder diagnosis**	**278**	**Yes**	**49 (17.6)**	**21 (31.3)**	**28 (13.3)**	**χ**^**2**^**=11.44; p=0.001**
		**No**	**229 (82.4)**	**46 (68.7)**	**183 (86.7)**	
Diagnosis of depression	278	Yes No	111 (33.9) 167 (60.1)	25 (37.3) 42 (62.7)	86 (40.8) 125 (59.2)	χ^2^=0.25; p=0.616
Diagnosis of bipolar disorder	278	Yes No	73 (26.3) 205 (73.7)	18 (26.9) 49 (73.1)	55 (26.1) 156 (73.9)	χ^2^=0.02; p=0.897
Diagnosis of schizophrenia	278	Yes No	17 (6.2) 261 (93.9)	5 (7.5) 62 (92.5)	12 (5.7) 199 (94.3)	χ^2^=0.28; p=0.567^1^
**Childhood trauma Questionnaire (CTQ) total score**	**264**	**Median (IQR)**	**38 (29-56)**	**47 (32-66)**	**36 (29-51)**	**Z=-2.89; p=0.004**
Intimate partner violence: score >3 on composite abuse scale (prior to admission)	248	Yes No	73 (29.4) 175 (70.6)	18 (33.3) 36 (66.7)	55 (28.4) 139 (71.7)	χ^2^=0.50; p=0.477
Intimate partner violence: total score on composite abuse scale (post-discharge)	243	Yes No	42 (17.3) 201 (82.7)	10 (19.2) 42 (80.8)	32 (16.8) 159 (83.3)	χ^2^=0.18; p=0.675
Highest service accessed	278	MBU Acute ward CRT	108 (38.9) 62 (22.3) 108 (38.9)	24 (35.8) 20 (29.9) 23 (34.3)	84 (39.8) 42 (19.9) 85 (40.3)	χ^2^=2.93; p=0.231
Total unmet needs CAN-M(S) (at one-month post-discharge)	278	Median (IQR)	3 (1-6)	4 (1-7)	3 (1-6)	Z=-0.93; p=0.352
CARE-Index (at one-month post-discharge)	201	Maternal sensitivity: Mean (SD) Maternal unresponsiveness: Mean (SD) Infant cooperation: Mean (SD) Infant passivity: Mean (SD)	4.0 (2.1) 7.2 (3.1) 3.0 (2.1) 5.0 (3.8)	4.5 (2.2) 6.9 (3.6) 3.4 (2.4) 5.4 (3.7)	3.9 (2.1) 7.3 (2.9) 2.9 (2.1) 4.9 (3.8)	t=-1.58; p=-.117 t=0.61; p=0.544 t=-1.41; p=0.159 t=-0.81; p=0.417
**PBQ scores (higher scores indicate more reported bonding problems at one-month post-discharge)**	**260**	**Median (IQR)**	**11 (5-19)**	**15 (9-30)**	**10 (4-17)**	**Z=-3.69; p=0.000**

All statistics are n (%) unless otherwise specified

^1^Fishers exact test used due to small cell sizes

Women who were re-admitted were also more likely to have received a personality disorder diagnosis (31.3% versus 13.3%; χ2 = 11.44, p = 0.001) and to have a higher total CTQ score indicating higher levels of childhood trauma (median score 47 versus 36; Z = -2.89, p = 0.004). CTQ scores were higher in the re-admitted group across all CTQ subscales, except for sexual abuse, with particularly high proportions in the re-admitted group meeting the cut-off for moderate/severe emotional abuse (46.0% vs 28.4%) or emotional neglect (42.9% vs 19.2%). Personality disorder and childhood trauma variables were highly correlated: 85% of women with a personality disorder diagnosis reported moderate/severe childhood trauma across at least one domain on the CTQ, and median CTQ scores were significantly higher for women with a personality disorder diagnosis (median = 56 versus 35; Z = -5.65, p = 0.000).

On the CAN-M(S), the most common unmet needs reported were difficulties with sleep (30.6%) and psychological distress (30.9%); however, there was no evidence of any difference in unmet needs by re-admission status. Ratings of observed mother–infant interactions on the CARE-Index suggested potential interaction difficulties post-discharge across both groups (e.g. mean scores for ‘maternal sensitivity’ were < 7 both among women who were and were not re-admitted). However, women who were later re-admitted self-reported more difficulties in their bond with their baby on the PBQ at one month post-discharge (median 15 versus 10; Z = -3.69; p = 0.000). Over a third (36.4%) of women who were re-admitted reported significant bonding difficulties on the PBQ (score > 25) at one month post-discharge compared with 15.1% of those not re-admitted.

### Variables associated with re-admission in the year after discharge

Given that receiving a personality disorder diagnosis and reporting childhood trauma were highly intercorrelated, we only included CTQ scores in our model: our research team, which included women with lived experience of perinatal mental health difficulties and childhood trauma, felt this was more representative of women’s self-reported experiences. Further analyses included univariable logistic regression models (Table 2, model 1) and a hierarchical logistic regression model (Table 2, model 2), with variables associated with re-admission input in steps: (Step 1) background maternal factors; (Step 2) clinical variables; (Step 3) continuing bonding difficulties post-discharge. Nagelkerke’s R-squared suggested that the model overall explained approximately 15% of the variability in re-admission rates.

**Table 2:** Factors associated with re-admission by 12-months post-discharge (complete case analysis)

			Model 1 Univariable (unadjusted)		Model 2 Multivariable					
					Step 1^i^ (n=251)		Step 2^ii^ (n=251)		Step 3^iii^ (n=251)	
	**Exposure**	N	Odds ratio (95% CI)	p-value	Odds ratio (95% CI)	p-value	Odds ratio (95% CI)	p-value	Odds ratio (95% CI)	p-value
1 Parental	Age	278	0.96 (0.92 – 1.01)	0.081	0.94 (0.89 – 1.00)	0.040*	0.94 (0.89 – 1.00)	0.039*	0.95 (0.90 – 1.00)	0.066
background	No further education	278	1.99 (1.05– 3.79)	0.036*	1.28 (0.59 – 2.79)	0.535	1.27 (0.58 – 2.77)	0.556	1.42 (0.64 – 3.18)	0.389
	Childhood trauma	264	1.02 (1.01 – 1.04)	0.001*	1.02 (1.01 – 1.04)	0.003*	1.02 (1.01 – 1.04)	0.009*	1.02 (1.00 – 1.03)	0.036*
2 Clinical factors	Previous admissions	278	2.22 (1.14 – 4.31)	0.019*			1.71 (0.78 – 3.76)	0.184	1.78 (0.80 – 3.97)	0.159
3 Bonding post-discharge	Postpartum bonding (PBQ)	260	1.04 (1.01 – 1.06)	0.001*					1.03 (1.01 – 1.06)	0.009*

*P<0.05

^i^Step 1: adjusting for background maternal factors (age, education, childhood trauma)

^ii^Step 2: adjusting for background maternal factors (age, education, childhood trauma) AND clinical variables (previous admissions in past 2 years)

^iii^Step 3: adjusting for background maternal factors (age, education, childhood trauma) AND clinical variables (previous admissions in past 2 years) AND continuing difficulties post-discharge (score on postpartum bonding questionnaire)

In Step 1, we found evidence that being younger (OR = 0.94; 95% CI 0.89–1.00, p = 0.040) and having a higher score for childhood trauma (OR = 1.02; 95% CI 1.01–1.04, p = 0.003) increased the odds of re-admission.

In Step 2, age (OR = 0.94; 95% CI 0.89–1.00, p = 0.039) and childhood trauma (OR = 1.02; 95% CI 1.01–1.04, p = 0.009) continued to show evidence of an association with re-admission once clinical history (previous admissions) had been included in the model as well. Adding this clinical variable into the model did not appear to improve the model’s fit (e.g. with previous admissions added to the model, the odds of re-admission continued to be 1.01–1.04 times higher for every unit increase on the CTQ scale of childhood trauma).

In the final step (Step 3), we found that reporting more difficulties in the mother–infant bond at one month post-discharge (i.e. having a higher score on the PBQ) was associated with a greater likelihood of re-admission (OR = 1.03; 95% CI 1.01–1.06, p = 0.009), after taking into account other covariates. Reporting a history of childhood trauma also continued to increase the odds of re-admission even after taking account of bonding difficulties (OR = 1.02; 95% CI 1.00–1.03, p = 0.036). Age showed weak evidence of an association (OR = 0.95; 95% CI 0.90–1.00, p = 0.066). In each case, effect sizes were small (Chen et al. 2010).

### Sensitivity analysis

In the multiple imputation analyses, results were broadly similar (Table 3). Reporting more difficulties in the mother–infant bond was associated with re-admission, after accounting for other covariates. A history of childhood trauma also independently increased the odds of re-admission.

**Table 3:** Factors associated with re-admission by 12-months post-discharge including multiple imputation of missing variables (N=279)

		Model 1 Univariable (unadjusted)		Model 2 Multivariable					
				Step 1^i^		Step 2^ii^		Step 3^iii^	
	**Exposure**	Odds ratio (95% CI)	p-value	Odds ratio (95% CI)	p-value	Odds ratio (95% CI)	p-value	Odds ratio (95% CI)	p-value
1 Parental	Age	0.96 (0.92 – 1.01)	0.081	0.97 (0.93 – 1.02)	0.242	0.97 (0.93 – 1.02)	0.250	0.98 (0.93 – 1.03)	0.352
background	No further education	1.99 (1.05– 3.79)	0.036*	1.68 (0.85 – 3.30)	0.134	1.61 (0.81 – 3.18)	0.171	1.81 (0.89 – 3.65)	0.100
	Childhood trauma	1.02 (1.01 – 1.04)	0.001*	1.02 (1.01 – 1.03)	0.002*	1.02 (1.01 – 1.03)	0.005*	1.02 (1.00 – 1.03)	0.025*
2 Clinical factors	Previous admissions	2.22 (1.14 – 4.31)	0.019*			1.89 (0.95 – 3.78)	0.070	1.97 (0.97 – 4.01)	0.060
3 Bonding post-discharge	Postpartum bonding (PBQ)	1.04 (1.02 – 1.06)	0.001*					1.03 (1.01 – 1.06)	0.003*

*P<0.05

^i^Step 1: adjusting for background maternal factors (age, education, childhood trauma)

^ii^Step 2: adjusting for background maternal factors (age, education, childhood trauma) AND clinical variables (previous admissions in past 2 years)

^iii^Step 3: adjusting for background maternal factors (age, education, childhood trauma) AND clinical variables (previous admissions in past 2 years) AND continuing difficulties post-discharge (score on postpartum bonding questionnaire)

## Discussion

In a cohort of 278 postpartum mothers admitted to acute psychiatric care in the year after childbirth, we found that almost a quarter (24.1%) were re-admitted to acute services within a year of discharge. The median number of further acute services accessed after discharge was two per woman, suggesting that a significant proportion of women experience a ‘revolving door’ of admissions over what is recognised as a critical period of child development (Stein et al. 2014).

Our study is the first of its kind to examine factors associated with re-admission after an initial postpartum admission. In univariable analyses, we found that being younger, less educated, having a history of prior admissions, experiences of childhood trauma, and reporting difficulties in the parent–infant bond one month after discharge all showed some evidence of an association with re-admission in the following year. In multivariable hierarchical logistic regression, a history of childhood trauma and reporting ongoing difficulties in the mother–infant bond one month after discharge continued to show evidence of independent associations with re-admission, after adjusting for socio-demographic and clinical covariates, though effect sizes were small. This was the case even though we did not find evidence of a difference in *observed* mother–infant interactions between mothers who were or were not re-admitted, although there were indications of potential interaction difficulties across both groups.

There is increasing evidence that bonding difficulties in the first two years of life are associated with long-term socio-emotional, cognitive, and physical difficulties in children (Ranson and Urichuk 2008; Winston and Chicot 2016) and our findings suggest that a mother’s perception of her bond with her baby may be particularly important in relation to her own mental health outcomes. Clinicians must therefore incorporate mothers’ perspectives when offering support. Our findings build on previous qualitative research which has found that women commonly report struggling with increased caregiving demands and responsibilities and a reduction in support after discharge from acute mental health treatment (Connerty et al. 2016; Griffiths et al. 2020). It is positive that, in England, the development of parent–infant support across community perinatal mental health services has become a key focus in the NHS Long Term Plan (NHS England 2019), as our findings suggest that some women have an unmet need for parent–infant support after discharge from acute services.

The transition to motherhood has long been seen as a time when adverse experiences in a mother’s own upbringing may also become particularly influential and distressing to cope with, and when trauma may in some cases pass intergenerationally from mother to infant in complex ways (Fraiberg et al. 1975). Our finding that childhood trauma is associated with repeated maternal psychiatric admissions adds weight to growing evidence of the long-term impact of childhood trauma on maternal mental health and well-being (Alvarez-Segura et al. 2014; McDonnell and Valentino 2016; NHS England 2021). A key implication is that services supporting women with perinatal mental health needs must be trauma-informed and develop appropriate assessments, care pathways, and interventions to ensure they are able to meet the needs of trauma survivors: currently these women appear to have poorer outcomes.

While perceptions of the mother–infant bond and having a history of childhood trauma were independently associated with higher odds of re-admission, past research also suggests that a mother’s perception of her baby and the mother–infant bond may be influenced by her own history of trauma (Christie et al. 2017; Fuchs et al. 2015). The potential that these factors may interact in complex ways and that trauma survivors may need additional support as they transition to motherhood would merit further investigation.

## Strengths and limitations

This is the first study to examine factors associated with re-admission among women admitted to acute psychiatric postpartum care. Detailed characterisations of women’s experiences were collected. However, only around 15% of variability in re-admission rates was explained by our model and a number of factors did not show evidence of influencing re-admission. This suggests that other factors, not measured in our study, are likely also to be important for re-admission. Future research should consider what additional challenges women may encounter in their lives after a postpartum admission. This could also include collecting information on support and treatment post-discharge and consideration of whether mothers’ needs differ depending on the infant’s age at the time of admission or discharge. Critically though, it should centre on asking women themselves what they believe influences their likelihood of future re-admissions.

While our study did examine factors such as whether mothers lived alone, and whether they had experienced recent domestic violence (neither of which increased the odds of re-admission in our cohort), we did not analyse social support in great depth in the current study. Future research could give greater consideration to the role of women’s support networks in relation to re-admission. Evidence suggests that social support becomes more important during critical transition periods like childbirth and can exert an increased influence on mental health (Bost et al. 2002; Robertson et al. 2004).

Finally, as over three-quarters of women in our study were from a White ethnic background, power was insufficient to assess potential differences in re-admission rates by ethnicity in detail. It would be valuable for future research to examine this in greater depth, given we know that women from ethnic minority backgrounds experience particular challenges accessing appropriate perinatal mental health care (Watson et al. 2019; Prady et al. 2016).

## Conclusion

Our study offers novel insights into factors associated with re-admission in the context of a postpartum psychiatric diagnosis. Our findings suggest that support with mother–infant bonding could be valuable to reduce re-admissions, along with greater consideration of the needs of mothers with a history of childhood trauma. However, effect sizes were small, and further research is needed to develop our understanding of what other factors may also influence re-admission rates in postpartum populations.

## Footnotes

1 Mother and baby unit was classified as the highest possible level of care, followed by acute ward, and then CRT. Where mothers had accessed more than one of these services, they were classified according to the highest service accessed (so that the CRT group had only accessed a CRT and no other acute service).

2 This was a composite variable comprised of whether the mother had (1) an unmet need for substance use recorded on the CAN-M outcome measure (see ‘Unmet needs post-discharge’ for description of this measure), or (2) a substance use disorder recorded on the International Classification of Diseases (ICD-10), or (3) substance use recorded on the Health of the Nations Outcome Scale routinely collected by services in England and Wales, or (4) reported substance use on the Smoking Alcohol and Drug use form (SAD).

## References

[CR1] Alvarez-Segura M, Garcia-Esteve L, Torres A, Plaza A, Imaz ML, Hermida-Barros L (2014). Are women with a history of abuse more vulnerable to perinatal depressive symptoms? A systematic review. Arch Womens Ment Health.

[CR2] Bernstein DP, Fink L (1998) Childhood trauma questionnaire: a retrospective self-report manual. Psychological Corporation, Orlando

[CR3] Bost KK, Cox MJ, Burchinal MR, Payne C (2002). Structural and Supportive Changes in Couples’ Family and Friendship Networks Across the Transition to Parenthood. J Marriage Fam.

[CR4] Brockington IF, Oates J, George S, Turner D, Vostanis P, Sullivan M et al (2001) A Screening Questionnaire for mother-infant bonding disorders. Arch Womens Mental Health 3(4):133–140

[CR5] Brockington IF, Fraser C, Wilson D (2006). The Postpartum Bonding Questionnaire: A validation. Arch Women’s Mental Health.

[CR6] Chen H, Cohen P, Chen S (2010) How big is a big odds ratio? Interpreting the magnitudes of odds ratios in epidemiological studies. Communications in Statistics - Simulation and Computation 39(4):860–864

[CR7] Christie H, Talmon A, Schafer SK, De Haan A, Vang ML, Haag K, et al (2017) The transition to parenthood following a history of childhood maltreatment: A review of the literature on prospective and new parents’ experiences. European Journal of Psychotraumatology. 2017;8(Suppl 7). 10.1080/20008198.2018.1492834 PMC7803084 33488997

[CR8] Connerty TJ, Roberts R, Sved WA (2016). Managing Life, Motherhood and Mental Health After Discharge from a Mother-Baby Unit: An Interpretive Phenomenological Analysis. Community Ment Health J.

[CR9] Crittenden P (2003). CARE-Index: Infants coding manual.

[CR10] Department of Health. Reference Guide to the Mental Health Act 1983 [Internet]. 2015. Available from: https://assets.publishing.service.gov.uk/government/uploads/system/uploads/attachment_data/file/417412/Reference_Guide.pdf

[CR11] Fraiberg S, Adelson E, Shapiro V (1975). Ghosts in the Nursery: A Psychoanalytic Approach to the Problems of Impaired Infant-Mother Relationships. J Am Acad Child Psychiatr.

[CR12] Fuchs A, Mohler E, Resch F, Kaess M (2015). Impact of a maternal history of childhood abuse on the development of mother-infant interaction during the first year of life. Child Abuse Negl.

[CR13] Griffiths LJ, Johnson RD, Broadhurst K, Bedston S, Cusworth L, Alrouh B (2020). Maternal health, pregnancy and birth outcomes for women involved in care proceedings in Wales: a linked data study. BMC Pregnancy Childbirth.

[CR14] Hegarty K, Null Fracgp, Bush R, Sheehan M (2005). The composite abuse scale: further development and assessment of reliability and validity of a multidimensional partner abuse measure in clinical settings. Violence Vict.

[CR15] Howard L, Hunt K, Slade M, O’Keane V, Seneviratne T, Leese M et al (2008) CAN-M: Camberwell assessment of need for mothers. The Royal College of Psychiatrists; 2008. 168

[CR16] Howard L, Trevillion K, Potts L, Heslin M, Pickles A, Byford S, Carson LE, Dolman C, Jennings S, Johnson S, Jones I, McDonald R, Pawlby S, Seneviratne G, Shallcross R, Stanley N, Wieck A, Abel K (2022a) Effectiveness and cost-effectiveness of psychiatric mother and baby units: Quasi-experimental study. BR J Psychiatry 1–9. 10.1192/bjp.2022.48 10.1192/bjp.2022.48 35505514

[CR17] Howard L, Abel K, Bick D, Bye A, Byford S, Carson L et al (2022b) Perinatal mental health services in pregnancy and the year after birth: the ESMI research programme including RCT. Programme Grants Appl Res. 10(5) 35700306

[CR18] Kendell RE, Chalmers JC, Platz C (1987). Epidemiology of puerperal psychoses. Br J Psychiatry.

[CR19] McDonnell CG, Valentino K (2016). Intergenerational Effects of Childhood Trauma: Evaluating Pathways Among Maternal ACEs, Perinatal Depressive Symptoms, and Infant Outcomes. Child Maltreat.

[CR20] Munk-Olsen T, Laursen TM, Pedersen CB, Mors O, Mortensen PB (2006). New parents and mental disorders: a population-based register study. JAMA.

[CR21] National Institute for Health and Care Excellence. Antenatal and postnatal mental health: clinical management and service guidance CG192 [Internet]. 2014. Available from: https://www.nice.org.uk/guidance/cg192 31990493

[CR22] Netsi E, Pearson RM, Murray L, Cooper P, Craske MG, Stein A (2018). Association of Persistent and Severe Postnatal Depression With Child Outcomes. JAMA Psychiat.

[CR23] NHS England. The NHS Long Term Plan [Internet]. England: NHS England; 2019. Available from: https://www.longtermplan.nhs.uk/wp-content/uploads/2019/08/nhs-long-term-plan-version-1.2.pdf

[CR24] NHS England (2021) A good practice guide to support implementation of trauma-informed care in the perinatal period. Available from: https://www.england.nhs.uk/publication/a-good-practice-guide-to-support-implementation-of-trauma-informed-care-in-the-perinatal-period/

[CR25] Parfitt Y, Pike A, Ayers S (2013). The impact of parents’ mental health on parent-baby interaction: a prospective study. Infant Behav Dev.

[CR26] Prady SL, Pickett KE, Gilbody S, Petherick ES, Mason D, Sheldon TA (2016). Variation and ethnic inequalities in treatment of common mental disorders before, during and after pregnancy: combined analysis of routine and research data in the Born in Bradford cohort. BMC Psychiatr.

[CR27] Ranson KE, Urichuk LJ (2008). The effect of parent-child attachment relationships on child biopsychosocial outcomes: A review. Early Child Dev Care.

[CR28] Robertson E, Grace S, Wallington T, Stewart DE (2004). Antenatal risk factors for postpartum depression: a synthesis of recent literature. Gen Hosp Psychiatry.

[CR29] Royal College of Psychiatrists. Perinatal Mental Health Services: Recommendations for the provision of services for childbearing women [Internet]. 2021. Available from: https://www.rcpsych.ac.uk/docs/default-source/improving-care/better-mh-policy/college-reports/college-report-cr232---perinatal-mental-heath-services.pdf?Status=Master&sfvrsn=82b10d7e_4

[CR30] Stein A, Pearson RM, Goodman SH, Rapa E, Rahman A, McCallum M (2014). Effects of perinatal mental disorders on the fetus and child. Lancet.

[CR31] Sterne JAC, White IR, Carlin JB, Spratt M, Royston P, Kenward MG (2009). Multiple imputation for missing data in epidemiological and clinical research: potential and pitfalls. BMJ.

[CR32] Trevillion K, Shallcross R, Ryan E, Heslin M, Pickles A, Byford S (2019). Protocol for a quasi-experimental study of the effectiveness and cost-effectiveness of mother and baby units compared with general psychiatric inpatient wards and crisis resolution team services (The ESMI study) in the provision of care for women in the postpartum period. BMJ Open.

[CR33] Wright T, Stevens S, Reed PW, Wouldes TA (2020). Post-discharge outcomes for mothers and the mother–infant relationship following admission to a psychiatric Mother-Baby Unit. Infant Ment Health J.

[CR34] Wittkowski A, Vatter S, Muhinyi A, Garrett C, Henderson M (2020). Measuring bonding or attachment in the parent-infant-relationship: A systematic review of parent-report assessment measures, their psychometric properties and clinical utility. Clin Psychol Rev.

[CR35] Winston R, Chicot R (2016). The importance of early bonding on the long-term mental health and resilience of children. London J Prim Care (abingdon).

[CR36] Watson H, Harrop D, Walton E, Young A, Soltani H (2019). A systematic review of ethnic minority women’s experiences of perinatal mental health conditions and services in Europe. PLoS ONE.

